# Emergence of a Clinical *Escherichia coli* Sequence Type 131 Strain Carrying a Chromosomal *bla*_*KPC–2*_ Gene

**DOI:** 10.3389/fmicb.2020.586764

**Published:** 2020-11-13

**Authors:** Dairong Wang, Xinli Mu, Ying Chen, Dongdong Zhao, Ying Fu, Yan Jiang, Yiwei Zhu, Jingjing Quan, Xiaoting Hua, Guofeng Mao, Xi Li, Yunsong Yu

**Affiliations:** ^1^Department of Infectious Diseases, Sir Run Run Shaw Hospital, College of Medicine, Zhejiang University, Hangzhou, China; ^2^Blood Center of Zhejiang Province, Hangzhou, China; ^3^Key Laboratory of Microbial Technology and Bioinformatics of Zhejiang Province, Zhejiang Institute of Microbiology, Hangzhou, China; ^4^Department of Clinical Laboratory, Sir Run Run Shaw Hospital, College of Medicine, Zhejiang University, Hangzhou, China; ^5^Department of Laboratory Medicine, Shaoxing People’s Hospital, Shaoxing, China; ^6^Centre of Laboratory Medicine, Zhejiang Provincial People’s Hospital, People’s Hospital of Hangzhou Medical College, Hangzhou, China

**Keywords:** E. coli, KPC-2, cre, resistance mechanism, whole genome sequencing

## Abstract

**Objectives:** Bacteria carrying the *Klebsiella pneumoniae* carbapenemase genes have rapidly spread worldwide and have become a great threat to public health. The *bla*_*KPC–2*_ gene has been primarily located on plasmids cocirculating in various strains. However, chromosomal integration of the *bla*_*KPC*__–2_ gene in *Escherichia coli* has not been reported. In the present study, we report the detection of the first clinical strain of *E. coli* ST131 with a *bla*_*KPC*__–2_ gene, which integrated in the chromosome. *E. coli* strain EC3385 was identified and subjected to susceptibility testing and genotyping. The complete genome sequences of this strain and four *Proteus mirabilis* strains were obtained. Chromosomal integration of the *bla*_*KPC–2*_ gene was confirmed using a combination of short- and long-read sequencing. Comparative genetic analyses were performed and the origin of the chromosomal location of the *bla*_*KPC–2*_ gene was further analyzed. Whole-genome sequencing revealed that strain EC3385 belonged to the ST131 type and possessed various resistance and virulence genes. Sequence analysis showed that the *bla*_*KPC–2*_ gene was carried in a 24-kb insertion sequence on the chromosome. This insertion sequence possessed high sequence similarity to previously reported *bla*_*KPC–2*_-habouring plasmids of *P. mirabilis* in China. To the best of our knowledge, this is the first report of a clinical ST131 *E. coli* strain carrying *bla*_*KPC–2*_ on the chromosome. The *bla*_*KPC–2*_ gene was probably horizontally transferred from the *P. mirabilis* plasmid to the *E. coli* chromosome by the *IS*26 element, indicating that *P. mirabilis* might be an important reservoir of *bla*_*KPC–2*_ gene for *E. coli*. Furthermore, the *E. coli* ST131 strain carrying the chromosomal *bla*_*KPC*__–2_ gene could be further spread due to its carbapenem resistance and high virulence. It is imperative to perform active surveillance to prevent further dissemination of KPC-2 type carbapenemase-producing isolates.

## Introduction

Bacteria carrying the *Klebsiella pneumoniae* carbapenemase genes (*bla*_*KPC*_) have rapidly spread worldwide and have become a great threat to public health because these bacteria are often associated with high morbidity and mortality (Wang et al., 2016; An et al., 2018). KPC-2 is the main type of KPC carbapenemase and is most common in *K. pneumoniae* bacteria. In China, clonal spreading is a main mode of transfer of KPC-2 type carbapenemase-producing *K. pneumonia.* Our previous research demonstrated that multilocus sequence type 11 (ST11) originated from a successful lineage of KPC-2 type carbapenemase-producing *K. pneumonia* in China (Qi et al., 2011).

In contrast to *K. pneumoniae*, *E. coli* strains have rarely been reported to carry the *bla*_*KPC–2*_ gene. However, recent reports found that the number of *E. coli* strains carrying the *bla*_*KPC–2*_ gene has increased. In addition, unlike *K. pneumoniae*, clonal spread has not been found for the *bla*_*KPC–2*_ gene of *E. coli* (Chen et al., 2014). These strains also have different clone types, such as ST131, ST410, ST2281, ST43, ST721, ST4385, and ST8 (Kim et al., 2012; Mavroidi et al., 2012; Tian et al., 2020). Notably, among these clone types, *E. coli* ST131, an international multidrug-resistant high-risk clone, has gained a further selective advantage as a result of acquiring carbapenem resistance (Rogers et al., 2011; Kim et al., 2012) and *E. coli* ST131 may become a successful lineage of KPC-2 type carbapenemase-producing *E. coli*.

In addition, *K. pneumoniae* carbapenemase genes have been primarily located on plasmids cocirculating with various strains (Nordmann et al., 2011). They are considered a major mechanism responsible for the dramatic increase in the prevalence of carbapenem-resistant *Enterobacteriaceae* isolates. Plasmid DNA can act as a temporary “lending library” allowing vital genes to survive various selective pressures (Harrison et al., 2015). Notably, *in vitro* data demonstrated that once a gene is incorporated into a chromosome, it is maintained through replication without being subject to selective pressures, and gene loss from bacterial populations is rare (Bergstrom et al., 2000; Bahl et al., 2009; Carraro et al., 2015). Interestingly, the earliest observed chromosomal *bla*_*KPC*_ gene integration events have been sporadic in gram-negative bacteria, such as *Pseudomonas aeruginosa* in 2006 (Villegas et al., 2007), *Raoultella spp.* in 2008 (Castanheira et al., 2009) and *Acinetobacter baumannii* in 2009 (Martínez et al., 2014). Recently, chromosomal integration has been described in four *K. pneumoniae* ST258 isolates (Conlan et al., 2014; Chen et al., 2015; Mathers et al., 2017). However, *bla*_*KPC*__–2_ gene chromosomal integration events in *E. coli* have not been reported.

In the present study, we report the detection of the first clinical strain of *E. coli* ST131 with a chromosomal *bla*_*KPC*__–2_ gene integrated in the chromosome. In addition, the genetic origin of this gene was further analyzed using whole-genome sequencing.

## Materials and Methods

### Patient and Strain Data

A patient was admitted to the hospital for a craniocerebral infarction in 2017. A carbapenem-resistant strain of *E. coli* EC3385 was isolated from sputum because the patient developed hospital-acquired pneumonia (HAP) secondary to postoperative intubation during the hospitalization. In addition, four *P. mirabilis* strains isolated at the same period (Table 1) as *E. coli* EC3385 in the ICU department were analyzed retrospectively. These strains were preliminary identified by the VITEK 2 system (Sysmex-bioMérieux, Marcy l’Etoile, France) and further confirmed by 16S rRNA sequencing.

**TABLE 1:** Strains collection date and Vitek-2 antibiotic susceptibility.

**Isolates**	**Collection day**	**MICs (mg/L)**											
		**AMK**	**CZA^*a*^**	**CRO**	**CST^*a*^ CIP**	**ETP**	**GEN**	**IPM^*a*^**	**LEV**	**TGC^*a*^**	**SXT**	**TGC**	**TZP**
*E. coli* EC3385	10-03-2017	≤2	0.25	≥64	0.25 ≥ 4	≥8	≤1	64	≥8	0.125	≤1/19	≤0.5	≥128
PM380	10-03-2017	≤2	0.125	≥64	- ≥ 4	≥8	≥16	64	≥8	-	≥16/304	-	64
PM906	21-03-2017	≤2	0.125	≥64	- ≥ 4	≥8	≥16	64	≥8	-	≥16/304	-	64
PM431	11-03-2017	≤2	0.125	≥64	- ≥ 4	≥8	≥16	64	≥8	-	≥16/304	-	64
PM187	08-02-2017	≤2	0.125	≥64	- ≥ 4	≥8	≥16	64	≥8	-	≥16/304	-	64
*E. coli* ATCC 25922	NA	≤2	≤0.125	≤1	0.125 ≤0.25	≤0.5	≤1	≤1	≤0.25	0.125	≤1/19	≤0.5	≤4

*^*a*^ Drug susceptibility was determined with broth microdilution method according to the Clinical Laboratory Standards Institute (CLSI) guidelines. NA, not applicable. AMK, amikacin; CZA, Ceftazidime-avibactam; CRO, ceftriaxone; CST, colistin; CIP, Ciprofloxacin; ETP, ertapenem; GEN, gentamicin; IPM, imipenem; LEV, levofloxacin; SXT, trimethoprim-sulfamethoxazole. TGC, Tigecycline, TZP, piperacillin-tazobactam.*

### Antibiotic Susceptibility Test

Antibiotic susceptibility was determined using the VITEK 2 system and broth microdilution method and the results were interpreted according to the Clinical and Laboratory Standard Institute (CLSI) guidelines (CLSI, 2017) except for tigecycline and colistin, which were interpreted according to the European Committee on Antimicrobial Susceptibility Testing breakpoints for *Enterobacteriaceae*^1^.

### Whole-Genome Sequencing and Assembly

Total genomic DNA extraction and analysis were performed as previously described (Li et al., 2018). Briefly, *E. coli* strain EC3385 and four *P. mirabilis* strains were cultured to mid-logarithmic phase in 50 ml of MH medium at 37°C. The genomic DNA of these strains was extracted using a QIAamp DNA MiniKit (Qiagen, Valencia, CA, United States) following the manufacturer’s recommendations. The DNA library was prepared using a Nextera XT DNA library preparation kit (Illumina, Inc., Cambridge, United Kingdom), and genomic DNA was sequenced on an Illumina HiSeq 4000 instrument with a 150-bp paired-end approach at a depth of approximately 200 ×. The raw reads of the strains were assembled into draft genomes using the CLC Genomics Workbench 10.0.

In addition, *E. coli* EC3385 strain sequencing was further performed via a single molecule real-time (SMRT) technique using a PacBio RS II platform and the resulting sequences were assembled *de novo* using the hierarchical genome assembly process (HGAP) with the default settings of the SMRT Analysis v2.3.0 software package (Shen et al., 2017).

### Genome Annotation and *in silico* Analyses

The Rapid Annotation using Subsystems Technology (RAST) annotation website server^2^ was used to annotate the genomes. Multi-locus sequence typing (MLST) of resistance genes and the Inc-type plasmid of the strain were performed using the MLST 1.8 server, ResFinder 3.0, Virulence Finder 1.5, and Plasmid Finder 1.3, which are available at the Center for Genomic Epidemiology^3^. Graphical maps were generated by the CGView server^4^. A comparison of the insert sequence of this strain and its related plasmids was performed with EasyFig 2.2.2 (Sullivan et al., 2011).

### Phylogenetic Analysis

Phylogenetic analysis of these *P. mirabilis* strains was performed. Genome sequences of other *P. mirabilis* strains were downloaded from the RefSeq database. Our strains were annotated by Prokka (Seemann, 2014) using the *P. mirabilis* proteins from the RefSeq database as a prior reference. The core genome was determined by Roary (Page et al., 2015) using Mafft for multiple sequence alignment. A maximum-likelihood phylogenetic tree was inferred by RAxML (Stamatakis, 2014) using the GTRGAMMA model for nucleotide substitution and running with 100 bootstraps. The phylogenetic tree was visualized by iTOL (Letunic and Bork, 2019).

### Nucleotide Sequence Accession Numbers

The complete nucleotide sequences of the chromosome and three plasmids of *E. coli* strain EC3385 reported in the present study were deposited in the GenBank nucleotide database under accession numbers CP029420, CP029421, and CP029422, respectively.

Sequence data from four *P. mirabilis* strains were also deposited in GenBank as follows:

CAV1042, CP018671.1; CAV1392, CP011578.1; CAV1453, CP018356.1.

## Results and Discussion

### Clinical Microbiologic Characteristics

The antimicrobial susceptibility test results showed that *E. coli* strain EC3385 was resistant to multiple antimicrobial agents, including cephalosporins, carbapenems and fluoroquinolones, but it was susceptible to aminoglycosides, ceftazidime-avibactam, colistin, and tigecycline (Table 1).

Multi-locus sequence typing analysis showed that this strain belonged to the ST131 type. The ST131-type *E. coli* clonal group emerged in the mid-2000s and has since spread extensively throughout the world (Can et al., 2015). Currently, the ST131 type is a very successful pandemic clone associated with community- and hospital-acquired infections. Many studies have demonstrated that this clone has high virulence potential and is associated with treatment failure (Can et al., 2015). In this study, VirulenceFinder analysis showed the presence of multiple potential virulence factors, such as *iss* (increased serum survival), *lpfA* (long polar fimbriae), and *gad* (glutamate decarboxylase) (Table 2). In addition, this clone is responsible for the rapid increase in β-lactam resistance among *E. coli*, mainly due to the production of CTX-M type extended spectrum β-lactamase enzymes (ESBLs) (Nicolas-Chanoine et al., 2014).

**TABLE 2:** Genome and plasmids of *E. coli* EC3385.

**Genomic structure**	**Size (bp)**	**GC content(%)**	**CDS no.**	**rRNA no.**	**tRNA no.**	**Accession no.**	**Resistance genes**	**Virulence genes**	**Incompatibility**
EC3385 chromosome	4,910,422	50.9	4749	66	267	CP029420	**^*bla*^**_*KPC–2*_	**^*iss*^**, **^*gad, lpfA, chuA*^^*fyuA, irp2, kpsMII_K5*^^*ompT, sitA, terC, traT*^, ^*Usp, yfcV*^**	–
EC3385-P1 plasmid	101,340	46.3	121	–	9	CP029421	–	–	incFIB
EC3385-P2 plasmid	89,323	50.5	132	–	–	CP029422	**^*bla*^**_*TEM–1B*_	–	incFIA

Interestingly, the isolate in this study did not carry additional genes encoding the CTX-M enzyme. A recent study reported that ESBL-negative ST131 strains have also been isolated worldwide (Ripabelli et al., 2020). In this study, no ESBL-encoding gene was detected in *E. coli* strain EC3385; instead, the *bla*_*KPC–2*_ gene, which encodes the KPC-2 type β-lactamase was identified by PCR amplification and sequencing.

### Chromosomal Integration of the *bla*_*KPC–2*_ Gene

*Escherichia coli* strain EC3385 carried the *bla*_*KPC–2*_ gene, which is primarily located on plasmids. However, further plasmid transfer and location experiments were not successful (data not shown), suggesting that the *bla*_*KPC–2*_ gene was located on the chromosome. Notably, a CTX-M type β-lactamase gene was found to be integrated in the chromosome of a high-risk *E. coli* ST131 clone by vertical transmission (Cerquetti et al., 2010; Stoesser et al., 2016), indicating that the ST131 type *E. coli* strain might have the ability to integrate resistance genes into chromosomes.

**FIGURE 1:**
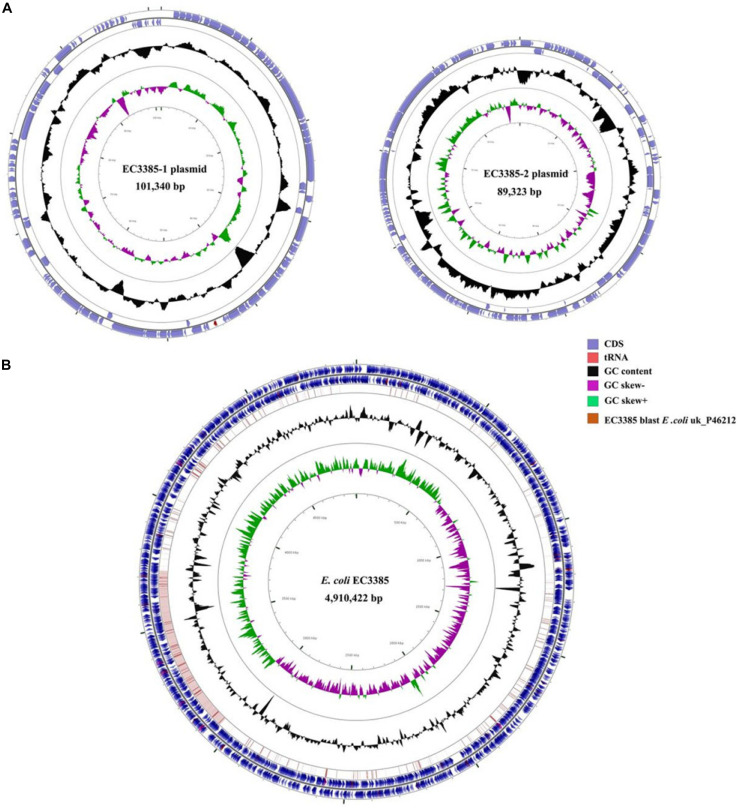
Circular maps of the *E. coli* EC3385 genome and its plasmids. **(A)** Circular graphs of two plasmids. **(B)** Circular graph of the EC3385 genome sequence and genome alignment. Blue arrows denote coding sequences, red arrows denote tRNA genes, and replication genes are denoted by green arrows. Genome alignment between EC3385 and *E. coli* uk_P46212 is shown in the outer circle in pink, and the GC content is shown in the inner circle in black. The region surrounding the *bla*_*KPC–2*_ gene is highlighted with a red frame.

To determine the gene location, whole-genome sequencing was performed. The whole-genome sequencing data were assembled, and a circular chromosome and two plasmids were generated (Table 2). The size of the genome was 4,910,422 bp, with a GC content of 50.9%, 66 rRNA operons, 267 tRNAs, and 4749 predicted protein-coding sequences (Table 2). Two plasmids approximately 89 to 101 kb in size and having a GC content between 50.5 and 46.3% were grouped into identifiable replicon types (Table 2 and Figure 1). Notably, the chromosomal location of the *bla*_*KPC–2*_ gene was determined using PacBio sequencing. Furthermore, the resequencing results further confirmed that the *bla*_*KPC–2*_ gene was located on the chromosome.

**FIGURE 2:**
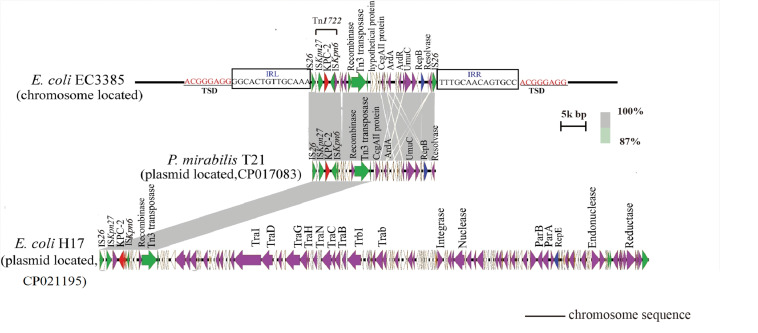
Linear comparison of the 24-kb insertion sequence with closely related plasmids. The gray regions between plasmids indicate nucleotide identity (87–100%) determined by BLASTn. Arrows indicate predicted open reading frames (ORFs). The primary structural characteristics of the 24-kb insertion sequence are compared to plasmids pT21 (CP017083) and pH17 (CP021195). Colored arrows represent ORFs, with red, purple, yellow, green, and white representing antibiotic resistance genes; replication, recombination and repair genes; plasmid stability genes; mobile elements and plasmid transfer related-genes; and genes with unknown function genes, respectively.

To evaluate the molecular basis of chromosomal integration, the chromosomal region encompassing the *bla*_*KPC–2*_ gene in the closed PacBio assembly of the ST131 type EC3385 isolate was aligned to reference the strain *E. coli* uk_P46212 (GenBank accession number CP013658), which belongs to the ST131 clone type. Relative to the reference, the EC3385 strain had a 24-kb insertion sequence in the chromosome, which included Tn1722 and several *IS*s (Figure 2).

The *bla*_*KPC–2*_ gene in the *E. coli* EC3385 strain was carried on this 24-kb composite transposon-like element flanked by two *IS*26 elements, which undergo replicative transposition with 8-bp target site duplication (TSD) (ACGGGAGG). This finding suggests the mobilization of this *bla*_*KPC–2*_ gene by the composite transposon formed by *IS*26 (Figure 2). *IS*26 has been demonstrated to undergo frequent intramolecular transposition. The structure of the insert sequence leads to the speculation that the *IS*26 element may facilitate recombination between the plasmid and chromosome (He et al., 2015).

A further BLAST search of the 24-kb insertion sequence against the GenBank database^5^ revealed that this sequence is highly similar to plasmid pT21 (GenBank accession no.CP017083), which was described in a KPC-2 type carbapenemase- producing *P. mirabilis* strain isolated in Zhejiang, China (Hua et al., 2020), with 99.9% query coverage and a maximum of 100% identity (Figure 2). In contrast, this 24-kb insertion sequence is only partly similar (47% query coverage and a maximum of 100% identity) to plasmid pH17-2 (GenBank accession no. CP021195) of a KPC-2 type carbapenemase-producing *E. coli* strain isolated in China (Figure 2; Zhao et al., 2018), indicating that capture of the chromosomal *bla*_*KPC–2*_ gene from *P. mirabilis* by plasmids is possible.

### Possible Origin of the Chromosomal *bla*_*KPC–2*_ Gene

**FIGURE 3:**
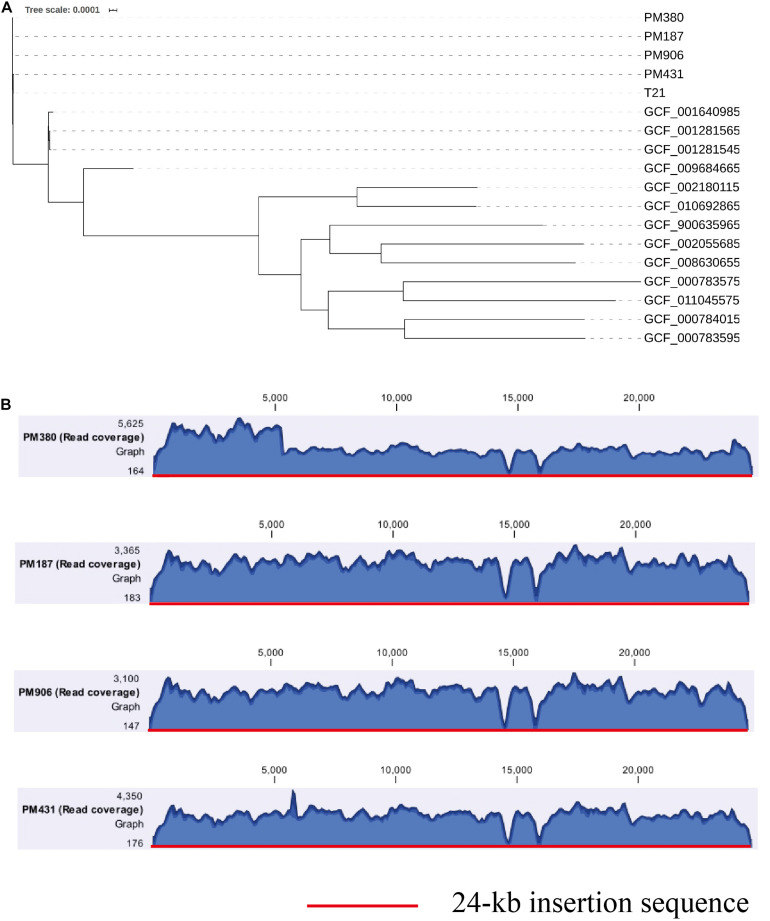
**(A)** A maximum-likelihood phylogenetic analysis of *P. mirabilis* strains. **(B)** Coverage of Illumina reads for four *P. mirabilis* strains (PM380, PM187, PM906, and PM431) mapped to the 24-kb insertion shown in Figure 2. The mean coverage for each strain is denoted by a curve (blue).

To further clarify the origin of the *bla*_*KPC–2*_ gene, four *bla*_*KPC–2*_-producing *P. mirabilis* strains isolated during the same period (approximately 2 months, Table 1) as *E. coli* EC3385 in the ICU department were analyzed retrospectively. These four *P. mirabilis* strains were all isolated from the sputum of different patients. Notably, a maximum-likelihood phylogenetic analysis between the four *P. mirabilis* strains and *P. mirabilis* T21 carrying the pT21 plasmid revealed that these strains were clustered together and belonged to the same clone (Figure 3A). Moreover, the whole-genome sequence analysis revealed that the four *P. mirabilis* strains all possessed a 24-kb insertion sequence (Figure 3B), indicating that this 24-kb insertion sequence that integrated into the chromosome of the *E. coli* EC3385 strain may have been acquired from *P. mirabilis* strains. In addition, two *P. mirabilis* strains were isolated before the *E. coli* EC3385 strain was identified, indicating that KPC-2 type carbapenemase-producing *P. mirabilis* strains may have spread in this ICU department. A limitation of this study is the lack of the direct links regarding the transmission between KPC-2 type carbapenemase- producing *P. mirabilis* and *E. coli* EC3385 strains. However, because the patients had stayed in the same department, it is most likely they were exposed to a common source.

## Conclusion

In summary, to the best of our knowledge, this is the first report of a clinical ST131 *E. coli* strain carrying the *bla*_*KPC–2*_ gene in the chromosome. The *bla*_*KPC–2*_ gene was probably horizontally transferred from the *P. mirabilis* plasmid to the *E. coli* chromosome by the *IS*26 mobile element, indicating that *P. mirabilis* might be an important reservoir of the *bla*_*KPC–2*_ gene for *E. coli*. Furthermore, the discovery of a chromosomal the *bla*_*KPC–2*_ gene in an *E. coli* strain is alarming. This gene will be maintained through replication without being subject to selective pressures, as the loss of chromosomal elements from bacterial populations is rare. Therefore, the *E. coli* ST131 strain carrying the *bla*_*KPC*__–2_ gene in the chromosome would be further spread due to its own carbapenem resistance and high virulence. It is imperative to perform active surveillance to prevent further dissemination of KPC-2 type carbapenemase-producing isolates.

## Data Availability Statement

The datasets presented in this study can be found in online repositories. The names of the repository/repositories and accession number(s) can be found in the article/supplementary material.

## Author Contributions

YY and XL conceived and designed the experiments. DW, XM, and YC performed the experiments. DZ, YZ, XH, GM, JQ, and YF analyzed the data. DW, XL, and YJ wrote the manuscript. All authors read and approved the final manuscript.

## Conflict of Interest

The authors declare that the research was conducted in the absence of any commercial or financial relationships that could be construed as a potential conflict of interest.

## Footnotes

1 http://www.eucast.org/clinical_breakpoints

2 http://rast.nmpdr.org/rast/cgi

3 http://www.genomicepidemiology.org/

4 http://stothard.afns.ualberta.ca/cgview_server/

5 http://blast.ncbi.nlm.nih.gov/Blast.cgi
